# Inhibition of autophagy promoted high glucose/ROS-mediated apoptosis in ADSCs

**DOI:** 10.1186/s13287-018-1029-4

**Published:** 2018-10-25

**Authors:** Qiang Li, Yating Yin, Yuqing Zheng, Feifei Chen, Peisheng Jin

**Affiliations:** 1 Department of Plastic Surgery, Affiliated Hospital of Xuzhou Medical University, Huai-hai West Road, Xuzhou, 221002 Jiangsu China; 2 Jiangsu Center for the Collaboration and Innovation of Cancer, Xuzhou Medical University, Huai-hai West Road, Xuzhou, 221002 Jiangsu China; 3 The Affiliated Friendship Plastic Surgery Hospital of Nanjing Medical University, Han-zhong Road, Nanjing, 210005 Jiangsu China

**Keywords:** Autophagy, Adipose tissue-derived stem cells, Apoptosis, High glucose

## Abstract

**Background:**

Increased apoptosis in adipose tissue-derived stem cells (ADSCs) limits their application in treating diabetes complications. Autophagy is a molecular process that allows cells to degrade and recover damaged macromolecules, and closely interacts with apoptosis. The aim of the present study was to investigate the potential role of autophagy in ADSC apoptosis induced by high glucose.

**Methods:**

Human ADSCs were cultured in normal or high-glucose medium for 6 h, 12 h, or 24 h. The effects of high glucose on ADSC autophagy, reactive oxygen species (ROS) production, and apoptosis were evaluated. The impact of autophagy on ROS production and apoptosis was explored by treatment with rapamycin or 3-methyladenine (3-MA). The c-jun kinase (JNK) signaling pathway was investigated by pharmacological disruption of SP600125.

**Results:**

ADSCs subjected to high glucose stress showed an obvious induction of autophagy and apoptosis and a significant increase in intracellular ROS levels. The JNK signaling pathway was confirmed to be involved in high glucose-induced autophagy. Pre-treatment with SP600125 or *N*-acetylcysteine reversed the effects of high glucose on the JNK signaling pathway and autophagy-related proteins. Pretreatment of ADSCs with 3-MA under high glucose stress induced a further increase in ROS levels compared to those of high glucose-treated cells. Furthermore, ADSCs pretreated with 3-MA under high glucose stress showed a marked increase in apoptosis compared with that of the cells treated with high glucose. Conversely, pre-treatment with rapamycin inhibited the apoptosis of ADSCs.

**Conclusions:**

Taken together, our data suggest that autophagy may play a protective role in high glucose-induced apoptosis in ADSCs. ROS/JNK signaling is essential in upregulating high glucose-induced autophagy. This study provides new insights into the molecular mechanism of autophagy involved in high glucose-induced apoptosis in ADSCs.

**Electronic supplementary material:**

The online version of this article (10.1186/s13287-018-1029-4) contains supplementary material, which is available to authorized users.

## Background

Diabetes is a chronic disease that affects over 347 million people globally. Due to diets with high fat and high sugar content accompanied by sedentary lifestyles, the global epidemic of diabetes is expected to rise [1, 2]. Diabetic patients are very susceptible to a myriad of complications, such as chronic wounds, cardiovascular damage, kidney failure, and diabetic foot disease, which lead to both patient morbidity and mortality [3–6]. Therefore, more efforts have been made to treat diabetes complications.

Adipose tissue-derived stem cells (ADSCs) are derived from adipose tissue stroma and have the ability to self-renew and differentiate into a number of functional cell types [7]. Emerging evidence has shown the beneficial effects of ADSC administration in treating various diseases because of the simple isolation techniques and easy scalability as well as the low immunogenicity and multipotency of ADSCs [8, 9]. Importantly, the use of ADSCs has been considered a novel tissue regenerative technique that has utility in diabetes complications [8, 9]. Unfortunately, increased apoptosis in stem cells limits their application in treating diabetes complications. A previous study demonstrated that high glucose results in the apoptosis of stem cells [10]. However, the intracellular mechanism of high glucose-induced ADSC apoptosis remains unclear.

Autophagy is the primary metabolic process by which eukaryotic cells degrade and recover damaged macromolecules and organelles [11]. During this process, substances in the cytoplasm are phagocytosed by autophagosomes, which are spherical structures with bilayer membranes, and transported to the lysosomes for degradation. The degradation products can be reused in the syntheses of macromolecules and in energetic metabolism. Autophagy is an important cell survival process [12, 13]. It has also been implicated in the cell death process [14]. Early studies suggested that autophagy serves as a cell survival mechanism in some pathological processes via its suppressive role in necroptosis and poly(ADP-ribose) polymerase (PARP)-mediated cell death during unfavorable growth conditions or cellular stress [15, 16]. In retinal ganglion cells, autophagy plays an important role in suppressing apoptosis, and it has been observed that activation of autophagy can promote retinal ganglion cell survival and that inhibition of autophagy can reduce cell survival during optic nerve degeneration [17]. In this study, we investigated the molecular mechanism of ADSC apoptosis and the changes in autophagic flux in high glucose-treated ADSCs to elucidate the role of autophagy in determining the fate of high glucose-treated ADSCs.

## Methods

### Isolation and culture of human ADSCs

All the methods were carried out as described in previous studies [18–20]. Adipose tissue samples were obtained from three liposuction aspirates of patients (age range 30–45 years) with informed consent at the Affiliated Hospital of Xuzhou Medical University. The tissues were washed with PBS and completely diced, and then all tissues were pooled and digested with 0.1% collagenase A (Sigma-Aldrich, St. Louis, MO, USA) solution at 37 °C for 60 min. The digested tissue was filtered using a 75-μm filter mesh (BD Biosciences, Franklin Lakes, NJ, USA) and centrifuged at 1200 rpm for 5 min, and the supernatant was removed along with the mature adipocytes. Subsequently, cell pellets were resuspended in L-Dulbecco’s modified Eagle’s medium (DMEM; Invitrogen, CA, USA) with 10% fetal bovine serum (FBS; Invitrogen, CA, USA) and cultured in flasks in an incubator with 5% CO_2_, at 37 °C. The medium was changed on the following day and every 3 days thereafter. When the cells reached 90% confluence, the cultures were trypsinized and passaged two more times. Passage 3–5 cells of all three donors were mixed equally and used as a pool for the following experiments.

### Cell treatment

ADSCs were cultured with normal glucose (5.5 mM) or high glucose (25 mM; Invitrogen, CA, USA) medium for 6, 12, or 24 h. 3-Methyladenine (3-MA, 5 mM, Sigma, St. Louis, MO, USA) or rapamycin (100 nM, Aladdin, Shanghai, China) was employed to pretreat cells for 24 h to inhibit or induce autophagy in ADSCs, respectively. An inhibitor of the c-jun kinase (JNK) signaling pathway, SP600125 (20 μg/ml, Beyotime, Shanghai, China) was used to pretreat the cells for 1 h. *N*-Acetylcysteine (NAC, 5 mM, Beyotime, Shanghai, China) was used to pretreat the cells for 1 h to inhibit ROS production in this study.

### Autophagy flux assay using mRFP-GFP-LC3

ADSCs were transfected with the tandem fluorescent-mRFP-GFP-LC3-adenovirus (HanBio, Wuhan, China), which expresses a specific marker of autophagosome formation to detect autophagy, according to the manufacturer’s instructions*.* The GFP signal is quenched in a lysosomal environment; in contrast, the RFP signal is more stable in an acidic environment [21]. Therefore, autophagosomes are labeled with yellow (green and red) or red. Five fields were chosen from three different cell preparations. GFP- and mRFP-expressing spots, which were indicated by fluorescent puncta and DAPI-stained nuclei, were counted manually.

### Measurement of intracellular ROS

Cells were seeded in a 6-well plate at a density of 5 × 10^4^ cells/well. The cells were added with 10 μM fluorescent probe CM-H2DCFDA (Molecular Probes) (Invitrogen, CA, USA) and incubated for 15 min at 37 °C in the dark. After washing with PBS, cells were harvested and analyzed using a FACS Calibur flow cytometer (BD Biosciences, Franklin Lakes, NJ, USA). To observe the degree of ROS production, cells were stained with 10 μM CM-H2DCFDA at 37 °C for 15 min, washed twice with PBS, and then analyzed by fluorescence microscopy (Olympus, Tokyo, Japan).

### Apoptosis assay

Following treatment, ADSCs were stained with fluorescein (FITC)-conjugated annexin V and propidium iodide (FITC/PI) (KeyGen Biotech, Nanjing, China) and analyzed on a flow cytometer to determine the rate of apoptosis.

A terminal deoxynucleotidy1 transferase-mediated dUTP nick end-labeling (TUNEL) assay (In Situ Cell Death Detection Kit; Roche Diagnostics) was also employed to determine the apoptosis of ADSCs. Briefly, ADSCs were incubated with TdT and fluorescein-labeled dUTP for 45 min at 37 °C. The percentage of apoptotic cells was then evaluated.

### Western blot analysis

Cell extracts were separated on SDS-polyacrylamide gels, and then the proteins were transferred to a nitrocellulose membrane and incubated with the rabbit polyclonal antibodies: anti-LC3B (1:500; Cell Signaling Technology, Danvers, MA, USA), anti-Beclin1 (1:500; Cell Signaling Technology), anti-ATG5 (1:500; Cell Signaling Technology), anti-caspase3 (1:500; Cell Signaling Technology), anti-cleaved-caspase3 (1:500; Cell Signaling Technology), anti-PARP (1:500; Cell Signaling Technology), anti-cleaved-PARP (1:500; Cell Signaling Technology), anti-JNK (1:500; Cell Signaling Technology), anti-p-JNK (1:500; Cell Signaling Technology), anti-AKT (1:500; Cell Signaling Technology), anti-p-AKT (1:500; Cell Signaling Technology), anti-ERK (1:500; Cell Signaling Technology), anti-p-ERK (1:500; Cell Signaling Technology), anti-p38 (1:500; Cell Signaling Technology), and anti-p-p38 (1:500; Cell Signaling Technology), as well as a mouse monoclonal antibody against β-actin (1:1000; Cell Signaling Technology). Immunoreactive protein bands were detected with Tanon scanning system (Tanon Science & Technology Co., Ltd., Beijing, China).

### Statistical analysis

The results are presented as the means ± S.D., and the data were statistically analyzed utilizing Student’s *t* test with SPSS software (SPSS 16.0, Inc., Chicago, IL, USA). *P* < 0.05 was considered as a statistically significant difference.

## Results

### High glucose induced autophagy in ADSCs

We first determined the stemness of the applied ADSCs by analysis of distinct surface markers in flow cytometry and analysis of osteogenic differentiation. The ADSCs presented a typical fibroblast-like morphology (Additional file 1: Figure S1A), which displayed positive staining for CD44 (98.1%), CD90 (98.2%), and CD105 (99.9%) and negative for CD31 (0.2%), CD34 (0.8%), and CD106 (1.7%) (Additional file 1: Figure S1B). The image of staining with Alizarin Red S indicated the presence of calcium deposition (Additional file 1: Figure S1C). The results demonstrated that the isolated ADSCs revealed typical ADSC characteristics.

**Fig. 1:**
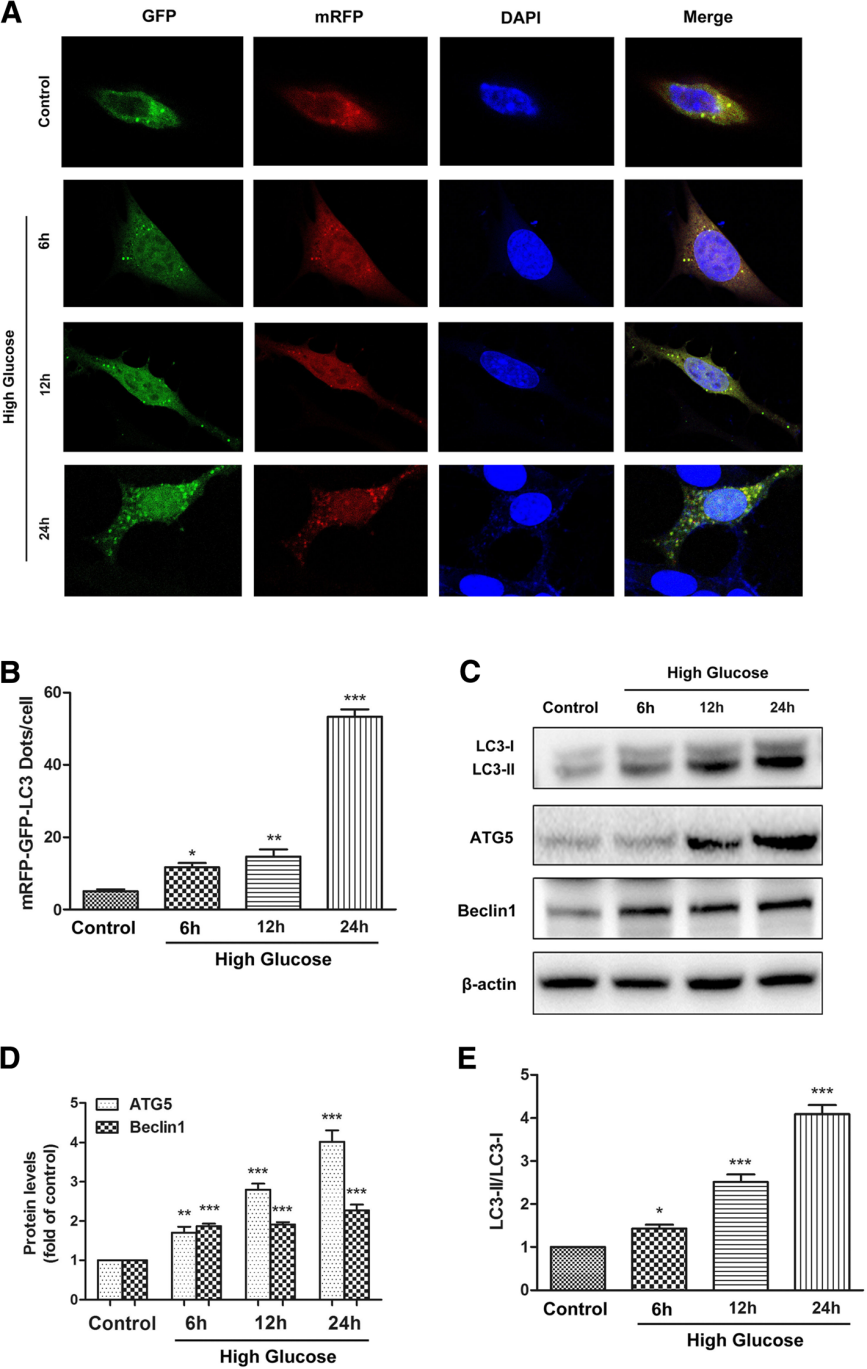
High glucose induced autophagy in ADSCs. ADSCs were cultured in high-glucose medium or normal-glucose medium for 6, 12, or 24 h. **a**, **b** Images showing the effects of high glucose on autophagy in fluorescent-mRFP-GFP-LC3-adenovirus-infected ADSCs. Green dots, autophagosomes; red dots, autolysosomes; yellow dots, autophagosomes. **c**–**e** Representative Western blot images showing the protein levels of LC3-I, LC3-II, ATG5, and Beclin1. β-actin was used as an internal control. Every experiment was repeated at least three times. Error bars indicate mean ± SD (*P < 0.05; **P < 0.01; ***P < 0.001)

Then, we investigated the impact of high glucose on autophagy in ADSCs. The autophagic flux was monitored by detecting and analyzing yellow and red fluorescent signals. As shown in the representative immunofluorescence images in Fig. 1a, b, the numbers of yellow and red puncta in the cells were significantly increased under high-glucose conditions in a time-dependent manner. LC3B distribution and the expression of the autophagy-associated genes ATG5 and Beclin1 were detected to characterize the autophagic flux. Western blot analysis revealed that high glucose significantly induced the expression of ATG5 and Beclin1 and the conversion of LC3-I to LC3-II, within 24 h (Fig. 1c–e). Collectively, these results suggested that high glucose stress significantly induced autophagy in ADSCs.

### High glucose promoted ROS generation in ADSCs

**Fig. 2:**
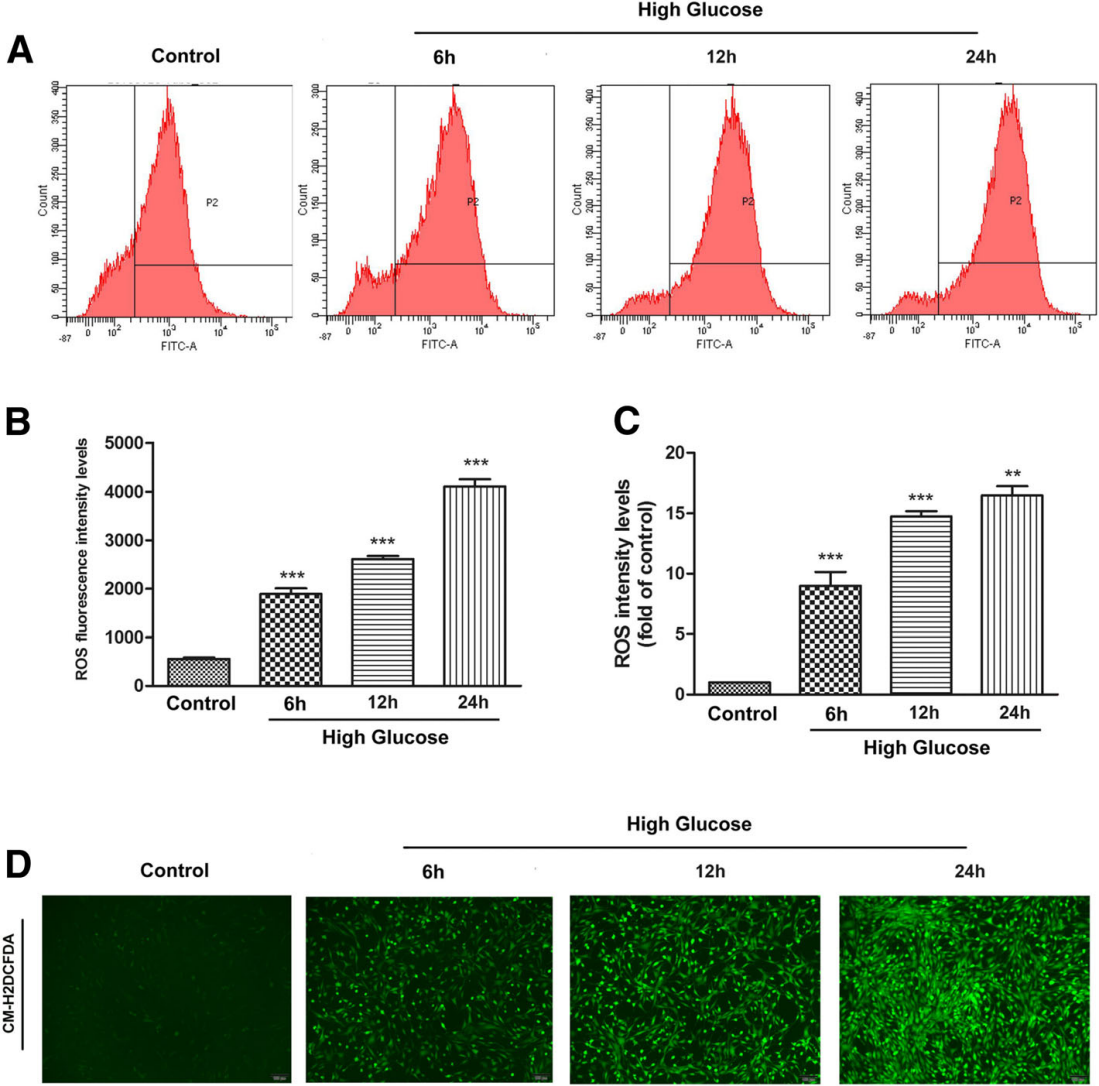
High glucose induced ROS generation in ADSCs. **a** Time course of ROS generation in ADSCs treated with high glucose. 5 × 10^4^ cells were incubated at 37 °C in the dark for 15 min with culture medium containing 10 μM CM-H2DCFDA to monitor ROS production. The degree of ROS production was measured by flow cytometer at an excitation/emission wavelength of 488 nm and 525 nm, respectively. **b** Quantification of ROS generation measured by flow cytometer in all groups. **c** Quantification analysis of the fluorescent intensity in all groups. **d** Intracellular ROS generation was visualized under the fluorescence microscope after cells were incubated with the fluorescent probe CM-H2DCFDA. Every experiment was repeated at least three times. Error bars indicate mean ± SD (**P < 0.01; ***P < 0.001)

Intracellular ROS play a vital role in different types of cell survival. We evaluated ROS levels via flow cytometry and fluorescence probe detection to explore the effect of high glucose on ROS generation. Data from the flow cytometer assay showed that high glucose promoted ROS generation in ADSCs in the time course (Fig. 2a, b). In addition, ADSCs cultured in high glucose stained with fluorescence probe were observed under a fluorescence microscope. The representative fluorescence results and the quantitative analysis revealed that high glucose promoted ROS generation in ADSCs (Fig. 2c, d).

### High glucose increased apoptosis in ADSCs

**Fig. 3:**
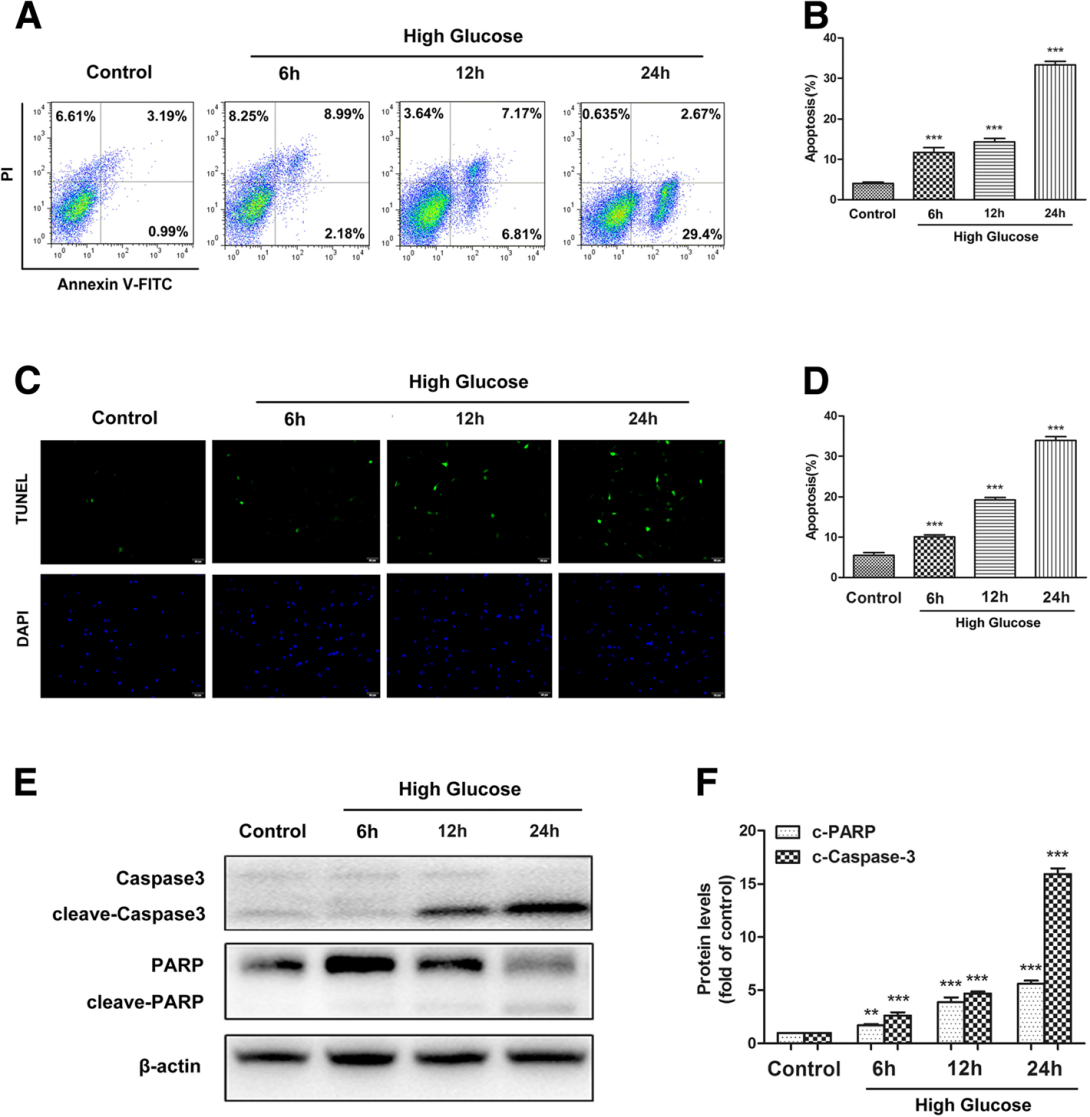
High glucose induced apoptosis in ADSCs. **a**, **b** ADSCs were under high glucose for 6, 12, and 24 h; cell apoptosis was detected using the annexin V-FITC/PI kit. Viable cells (annexin V^−^/PI^−^), early apoptotic cells (annexin V^+^/PI^−^), late apoptotic cells (annexin V^−^/PI^+^), and necrotic cells (annexin V^+^/PI^+^) are located in the bottom left, bottom right, and top right quadrants, respectively. The numbers in each quadrant represent the percentage of cells. The early apoptotic cells (annexin V^+^/PI^−^) and late apoptotic cells (annexin V^−^/PI^+^) were analyzed. **c**, **d** Representative TUNEL staining imaging of ADSCs under high-glucose conditions for 6, 12, or 24 h. **e**, **f** Western blot analysis of protein levels of cleaved-caspase-3 and PARP. β-actin was used as an internal control. The data are representative of three independent experiments. Error bars indicate mean ± SD (**P < 0.01; ***P < 0.001)

To investigate the apoptosis of ADSCs induced by high glucose stress, we treated cells with high glucose for different periods of time and then evaluated cell apoptosis by flow cytometry. The results showed that high glucose induced cell apoptosis in a time-dependent manner (Fig. 3a, b). TUNEL staining assay was also employed to examine ADSC apoptosis under high-glucose conditions. The representative TUNEL results show and the quantitative analysis demonstrated that high glucose increased the apoptosis of ADSCs in a time-dependent manner (Fig. 3c, d). Caspase3 and poly(ADP-ribose) polymerase (PARP) are principal apoptosis markers through which the mitochondrial and cytosolic pathways induce apoptosis (Additional file 2). Consequently, we examined the expression of cleaved-caspase3 and PARP. Western blot analysis indicated that high-glucose treatment promoted cleaved-caspase3 and PARP expression (Fig. 3e, f). Taken together, these results indicated that high glucose stress significantly induced the apoptosis of ADSCs.

### The ROS-mediated JNK signaling pathway was involved in high glucose-induced autophagy in ADSCs

**Fig. 4:**
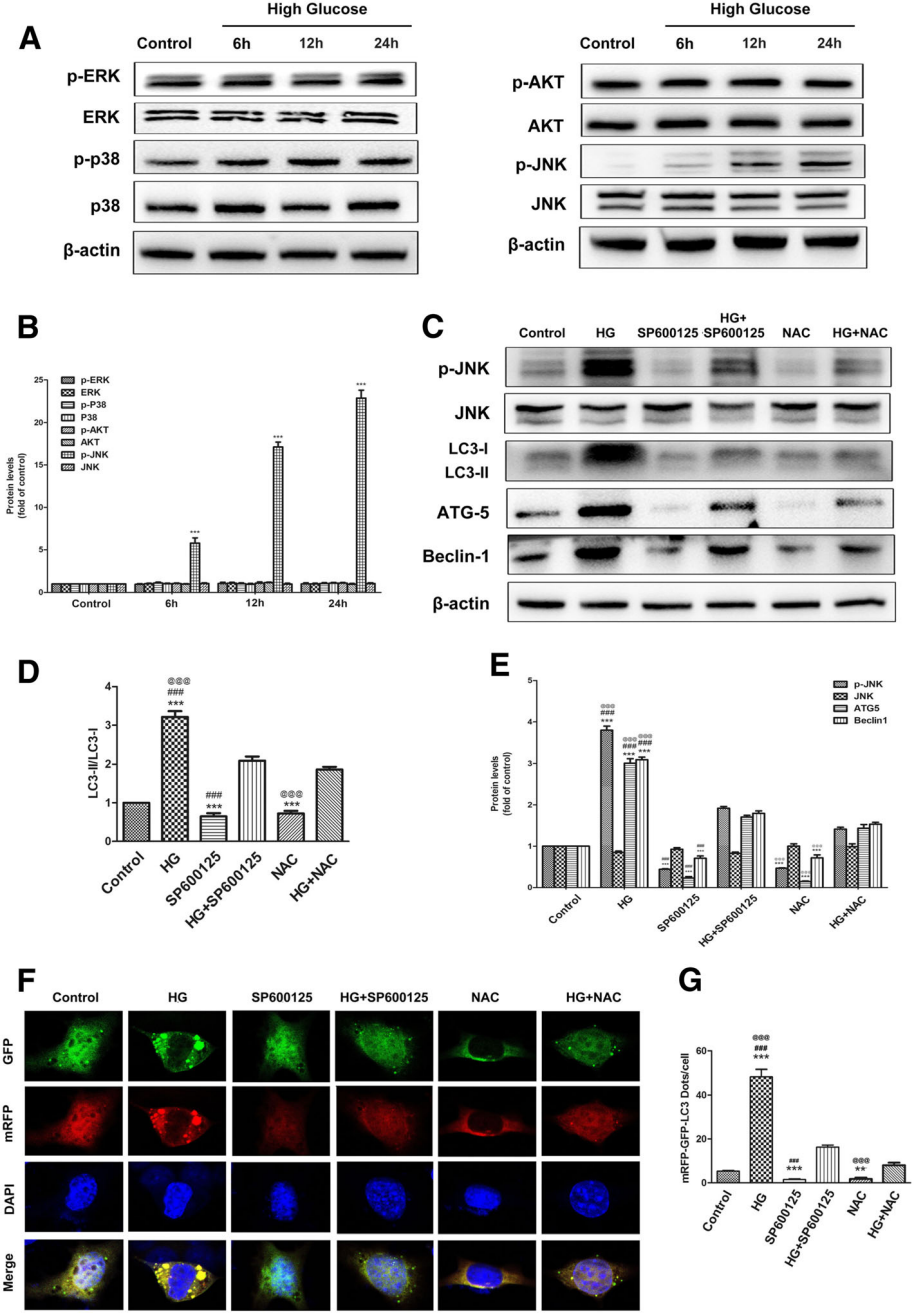
High glucose-induced autophagy was mediated by the ROS/JNK signaling pathway. **a**, **b** Time course analysis of phosphorylated and total p38, ERK, JNK, and AKT protein expression in ADSCs cultured in high-glucose condition. β-actin was used as an internal control. **c**–**e** Cells were pre-treated with SP600125 (20 μg/ml) or NAC (5 mM) for 2 h and then cultured in high glucose for 24 h. Western blot analysis of JNK, p-JNK, LC3B, Becline-1, and ATG5 expression. **f**, **g** Cells were infected with mRFP-GFP-LC3 adenovirus. Confocal microscopic analysis is shown. Green dots, autophagosomes; red dots, autolysosomes; yellow dots, autophagosomes. The data are representative of three independent experiments. Error bars indicate mean ± SD (*P < 0.05, **P < 0.01, ***P < 0.001 vs control; ^###^*P* < 0.001 vs high glucose+SP600125; ^@@@^*P* < 0.001 vs high glucose+NAC)

Autophagy activity is tightly controlled by the serine/threoninekinase (AKT) and mitogen-activated protein kinase (MAPK) pathways [22, 23]. To investigate whether high glucose triggers the MAPK and AKT pathways in ADSCs, we first detected the phosphorylation of MAPK family members (ERK1/2, JNK, and p38) and AKT signaling activity after high glucose stress. As shown in Fig. 4a, b, high glucose significantly increased JNK activation in a time-dependent manner, but there was no change in ERK1/2 and p38 phosphorylation and AKT activity. There is evidence that the ROS-mediated JNK pathway is responsible for the induction of autophagy [24]. Therefore, we explored the effect of the ROS/JNK signaling pathway on autophagy levels in high glucose-treated cells. We investigated the activity of the JNK signaling pathway and the autophagy process in the absence or presence of the JNK inhibitor SP600125 or the ROS inhibitor NAC using Western blot analysis. The results showed that high glucose increased the expression of p-JNK, ATG5, and Beclin1. In addition, the conversion of LC3I to LC3II was concomitantly increased. However, pretreatment with SP600125 or NAC before high-glucose treatment reversed the effect of high glucose on the JNK signaling pathway and autophagy-related proteins (Fig. 4c, d). Moreover, we monitored the autophagic flux via adenovirus infection. The representative immunofluorescence images show that the increased numbers of yellow and red puncta induced by high glucose were significantly decreased by SP600125 or NAC (Fig. 4e, f). These results revealed that the ROS-mediated JNK signaling pathway was involved in high glucose-induced autophagy in ADSCs.

### Autophagy had a potential role in reducing ROS generation in high glucose-treated ADSCs

**Fig. 5:**
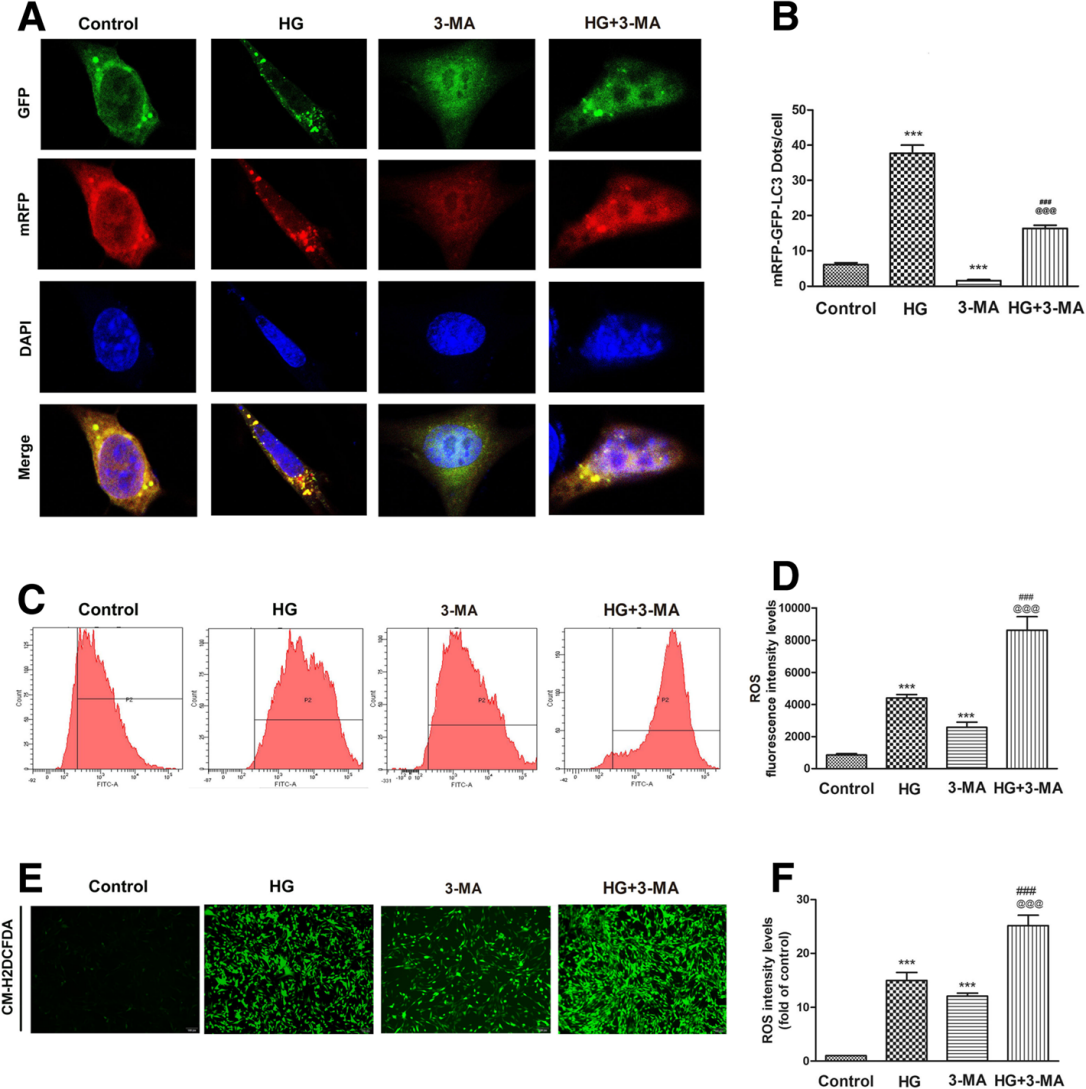
Inhibition of autophagy promoted high glucose-induced ROS production in ADSCs. **a**, **b** Cells were pre-treated with 3-MA for 24 h and then cultured in high-glucose medium for 24 h. Cells were infected with mRFP-GFP-LC3 adenovirus and observed under confocal microscope. Green dots, autophagosomes; red dots, autolysosomes; yellow dots, autophagosomes. **c**, **d** The level of DCF-sensitive ROS was measured by a flow cytometer. **e**, **f** Intracellular ROS generation was visualized under the fluorescence microscope. The scale bars represent 100 μm. Data are shown as means ± S.D. of three independent replicates (***P < 0.001 vs control; ^###^*P* < 0.001 vs high glucose; ^@@@^*P* < 0.001 vs 3-MA)

Autophagy can remove damaged mitochondria and reduce ROS production. We explored the effect of inhibiting autophagy on the potential high glucose-mediated ROS production to determine the biological significance of autophagy in ADSCs in response to high glucose. We first used 3-MA to inhibit autophagy for 24 h. Then, cells were infected with fluorescent-mRFP-GFP-LC3-adenovirus. The immunofluorescence images show that the numbers of yellow and red puncta in the cells were significantly increased by high glucose. This effect was reversed when 3-MA was given (Fig. 5a, b). Next, we employed flow cytometry and fluorescence probe detection to determine the role of autophagy in regulating ROS generation in high glucose-treated ADSCs. We found that autophagy inhibition induced a further increase in ROS levels when compared to the levels in high glucose-treated cells (Fig. 5c–f). Taken together, these data suggest that autophagy might play an essential role in reducing ROS generation in high glucose-treated ADSCs.

### Autophagy regulated the apoptosis of ADSCs induced by high glucose

**Fig. 6:**
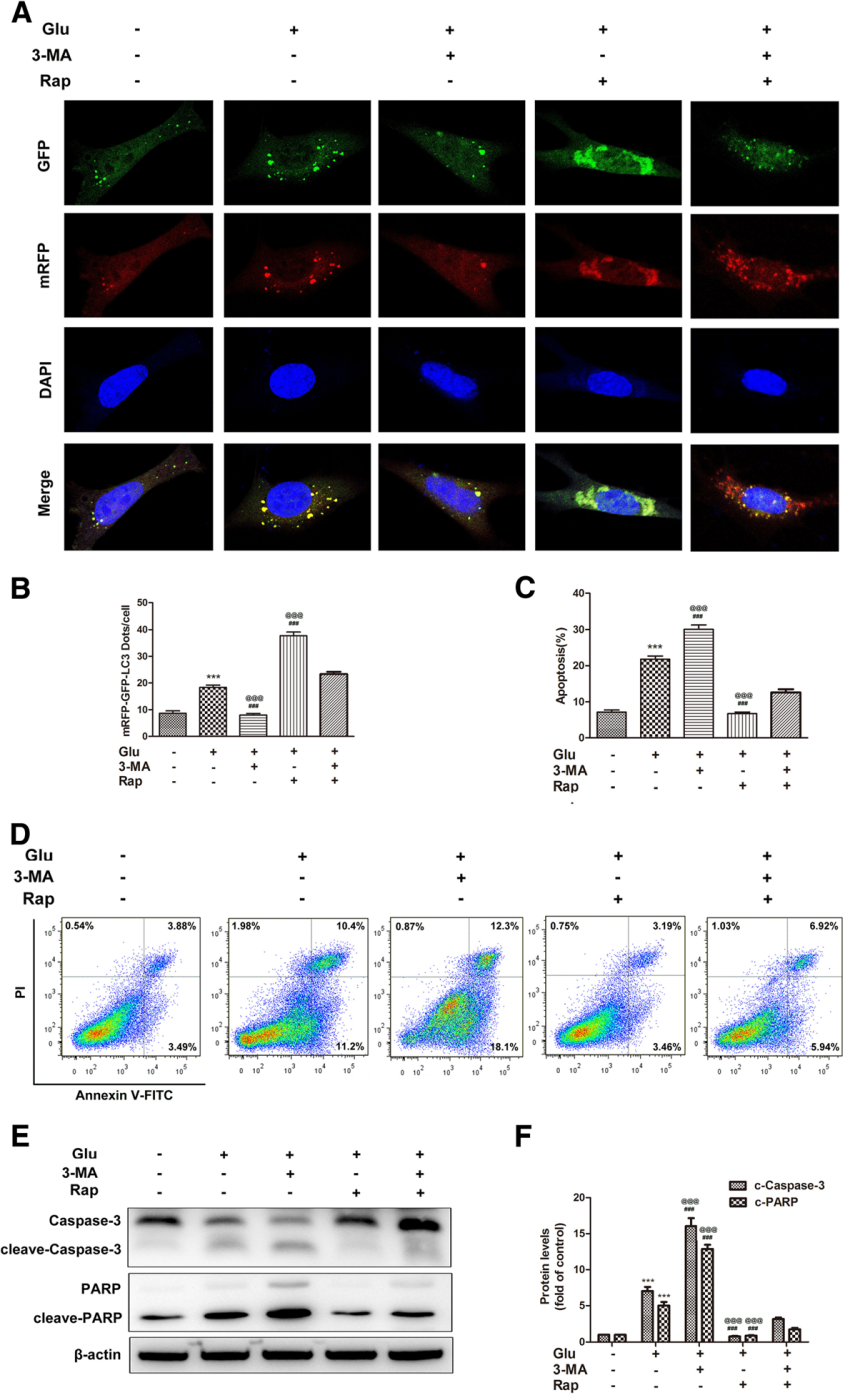
Inhibition of autophagy aggravated high glucose-induced apoptosis of ADSCs. **a**, **b** Cells were pre-treated with 3-MA (5 mM) or rapamycin (100 nM) for 24 h and then cultured in high glucose for 24 h. Cells were infected with mRFP-GFP-LC3 adenovirus and observed under confocal microscope. Green dots, autophagosomes; red dots, autolysosomes; yellow dots, autophagosomes. **c**, **d** ADSCs were stained with annexin V-FITC/PI and immediately analyzed by flow cytometry using the annexin V-FITC/PI kit. The early apoptotic cells (annexin V^+^/PI^−^) and late apoptotic cells (annexin V^−^/PI^+^) were analyzed. **e**, **f** The protein levels of cleaved-caspase-3 and cleaved-PARP and β-actin were determined by Western blot analysis. Each value is expressed as the mean ± SD of three independent experiments (***P < 0.001 vs control; ^###^*P* < 0.001 vs high glucose; ^@@@^*P* < 0.001 vs high glucose+3-MA+rapamycin)

Autophagy represents a double-edged sword in cell fate. To gain insight into the role of autophagy in the apoptosis induced by high glucose, we used rapamycin and 3-MA to regulate autophagy in ADSCs. Figure 6a, b shows that rapamycin promoted high glucose-induced autophagy while 3-MA reversed this effect. Then, we employed a flow cytometry assay to detect apoptotic cell death and determined the role of autophagy in high glucose-mediated apoptotic cell death. The results showed that rapamycin inhibited the apoptosis of ADSCs induced by high glucose, while 3-MA induced cell apoptosis (Fig. 6c, d). Furthermore, we determined the levels of the apoptosis-related molecules cleaved-caspase3 and PARP by Western blot analysis. Compared with that in high glucose-treated ADSCs, rapamycin inhibited the cleavage of caspase3 and PARP while 3-MA increased cleaved-caspase3 and PARP expression (Fig. 6e, f). Collectively, these data suggest that autophagy has a protective effect on high glucose-induced apoptosis in ADSCs.

## Discussion

Although recent studies have shown beneficial effects of ADSC administration in various diseases, impairment of resident and recruited cell functions due to disease complications strongly delays such effects in the treatment of diabetes. Extensive research has shown that ADSC apoptosis is an important pathophysiological event in various complications of diabetes [25, 26]. However, the underlying mechanism has not been fully elucidated.

**Fig. 7:**
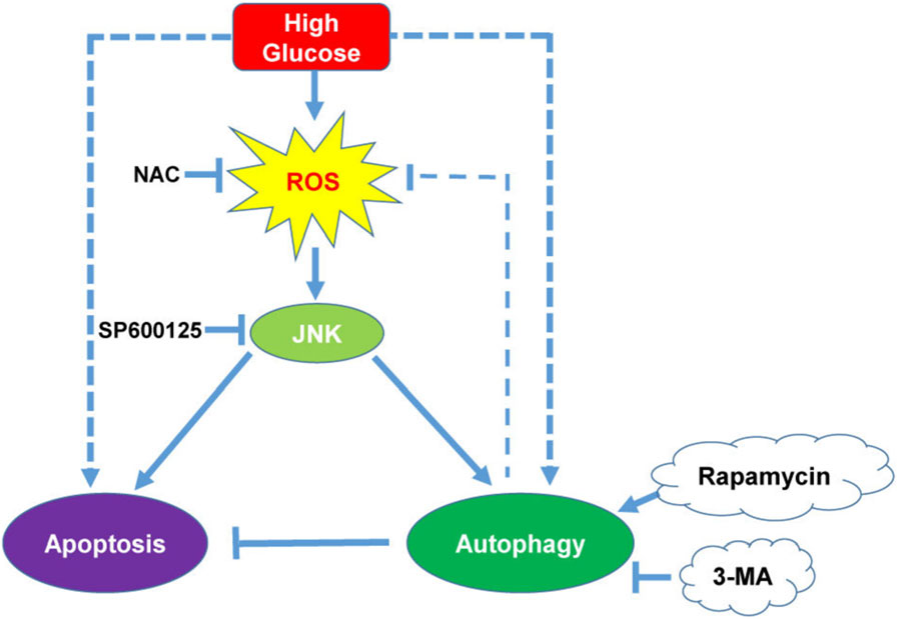
Schematic diagram of how autophagy prevented high glucose/ROS-mediated apoptosis

Autophagy, the basic catabolic process, occurs at basal levels in most tissues and contributes to routine cell recycling by lysosomes. In addition to the turnover of unnecessary or dysfunctional cellular components, autophagy is also involved in the development and differentiation of certain human diseases [11, 21]. There is abundant evidence indicating that high glucose increases autophagy in different cell types. However, the effect of high glucose on autophagy in ADSCs is unknown. We used a tandem fluorescent-mRFP-GFP-LC3-adenovirus to examine autophagy. The data showed that autophagic flux was significantly increased under high-glucose conditions. Beclin-1 interacts with several cofactors to promote the formation of Beclin-1-Vps34-Vps15 core complexes, which initiate autophagy [27]. When autophagy is initiated, LC3-I could be converted to LC3-II which incorporated into autophagic vacuoles [28]. In addition, ATG5 is an important protein associated with phagophore formation, and deletion of ATG5 results in the complete absence of LC3-II [29]. Thus, we employed these markers to explore the effect of high glucose stress on the level of autophagy in ADSCs. Our data showed that high glucose increased Beclin1, and ATG5 levels and LC3-I/LC3-II conversion. High glucose has been reported to induce autophagy in cardiac cells and human nucleus pulposus cells. In the present study, our results confirmed the ability of high glucose to induce the autophagic response in ADSCs (Fig. 7).

ROS are highly reactive oxygen free radicals or nonradical molecules that have essential roles in deciding cell fate [30, 31]. A hypothesis has been proposed that high blood glucose induces oxidative stress through the generation of excessive ROS, which play a dominant role in the development of chronic complications caused by diabetes [32, 33]. Several studies have suggested that high glucose can lead to the accumulation of ROS in endothelial cells and initiate apoptosis [34]. Peroxisome proliferator-activated receptor-γ coactivator 1α (PGC-1α) is an important mediator of the metabolic effects of ROS, as PGC-1α activation results in increases in mitochondrial energy metabolism and the cellular capacity to detoxify ROS, thereby reprogramming cell metabolism to maintain survival [35, 36]. In this study, we explored the effect of high glucose on ROS production and found that high glucose induced ROS generation. Moreover, the number of apoptotic cells increased after culture under high-glucose conditions.

Under normal physiological conditions, the ROS level is maintained within a certain range due to the balance between ROS production and scavenging. However, under some pathological conditions, this balance can be broken and can lead to ROS generation. It is well known that enhanced ROS production induces autophagy [37]. To confirm our preceding findings showing the relationship between ROS and autophagy, we used NAC, an ROS scavenger, to treat the cells before high glucose exposure. We found that NAC could successfully reverse high glucose-induced autophagy and the expression of autophagy-related markers, suggesting that high glucose-induced autophagy was related to ROS accumulation in ADSCs. As previously reported, autophagy can eliminate increased mitochondria after damage in several tissues, including liver, muscle, and neuronal tissue [22, 38, 39]. The next question is whether autophagy can eliminate damaged and ROS-producing mitochondria to protect ADSCs under conditions of high glucose. We used 3-MA to inhibit autophagy and we found that inhibition of autophagy induced a further increase in ROS levels compared to the levels in high glucose-treated cells. These findings further demonstrated the relationship between oxidative stress and autophagy and suggested that autophagy mediates its protective effects by suppressing ROS accumulation.

MAPK signals are frequently overactivated in a variety of disease states. It has been reported that ROS regulate the MAPK signaling pathway to modulate autophagy [23]. Additionally, many lines of evidence suggest that activation of the AKT signaling pathway is responsible for ROS-triggered autophagy in cells [40]. To investigate whether MAPK or AKT signals are involved in regulating the effect of high glucose on autophagy, we first detected the levels of the phosphorylated forms of MAPK family members or AKT after treatment with high glucose. We found that the JNK signaling pathway was activated by high glucose. The JNK inhibitor SP600125 reversed the effect of high glucose on ADSC autophagy. Interestingly, NAC inhibited JNK phosphorylation. These results revealed that the JNK signaling pathway, which triggers autophagy, functions as a downstream signal of ROS in high glucose-treated ADSCs.

There is evidence that autophagy is an essential and homeostatic process by which cells break down their own components, thus escaping apoptosis induced by diverse stress conditions [11, 21]. A previous study demonstrated that suppression of autophagy was protective in high glucose-induced cardiomyocyte injury [41], suggesting that autophagy precedes apoptosis as a defense mechanism to reestablish homeostasis. Conversely, other studies have revealed that enhanced cardiac autophagy protects against cardiomyocyte apoptosis in diabetes, indicating that autophagy is a protective process against high glucose injury [42]. The detailed physiological effects of autophagy are still debatable. In the current study, we used 3-MA and rapamycin to regulate the autophagy level in ADSCs. We found that high glucose-induced apoptosis in ADSCs was abolished by autophagy upregulation, whereas the apoptosis was aggravated by autophagy inhibition. Therefore, it is apparent that high glucose-triggered autophagy may regulate cytoprotective effects in ADSCs.

## Conclusions

In summary, our studies provide evidence that ROS play an important role in regulating high glucose-induced apoptosis and autophagy in ADSCs. Moreover, we identified a protective role of autophagy and ROS generation and cell apoptosis was markedly enhanced when autophagy was inhibited. These results suggest that targeting autophagy in ADSCs might be a potential therapeutic strategy for diabetes complications.

## Additional files

Additional file 1: Characteristics of ADSCs. (PDF 1563 kb)

Additional file 2: Supplementary materials and methods. (PDF 56 kb)

## Abbreviations

3-MA: 3-Methyladenine
ADSCs: Adipose tissue-derived stem cells
AKT: Serine/threoninekinase
DMEM: L-Dulbecco’s modified Eagle’s medium
ERK: Extracellular regulated protein kinases
FBS: Fetal bovine serum
JNK: c-jun kinase
MAPK: Mitogen-activated protein kinase
NAC: *N*-Acetylcysteine
PARP: Poly(ADP-ribose) polymerase
PGC-1α: Peroxisome proliferator-activated receptor-γ coactivator 1α
ROS: Reactive oxygen species
