# Supplementary figures and images for: Inhibition of autophagy promoted high glucose/ROS-mediated apoptosis in ADSCs
Source: Stem Cell Res Ther. 2018 Oct 25;9:289. doi: 10.1186/s13287-018-1029-4 (PMC6203262; doi:10.1186/s13287-018-1029-4)

**A**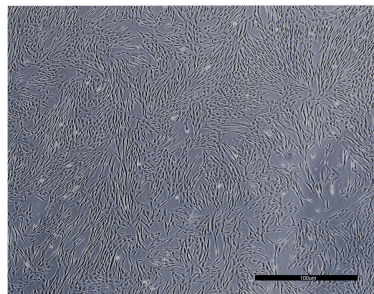**B**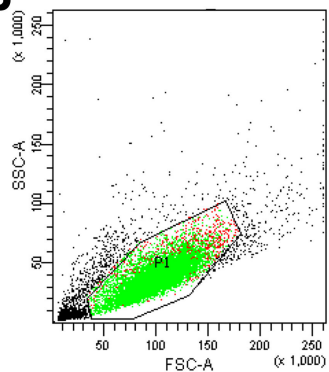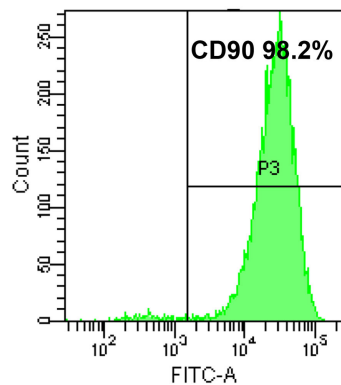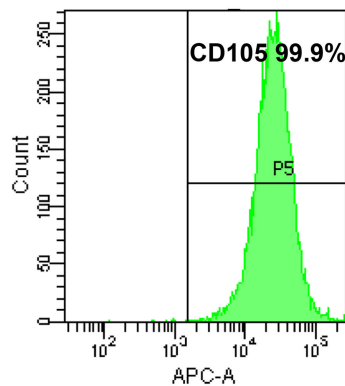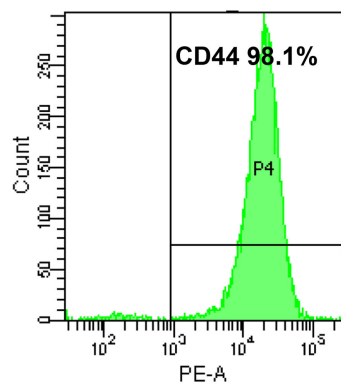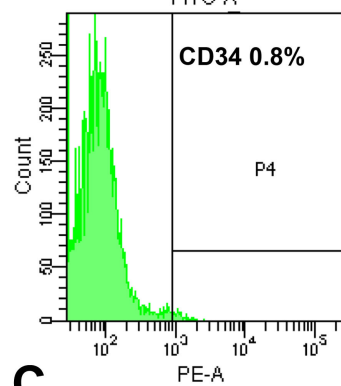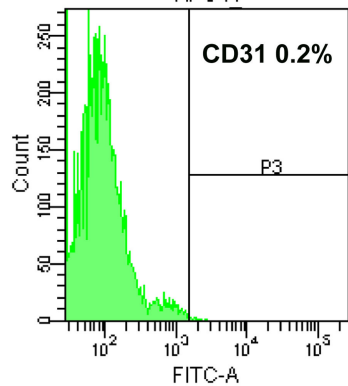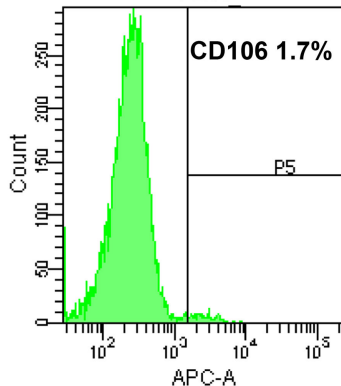**C**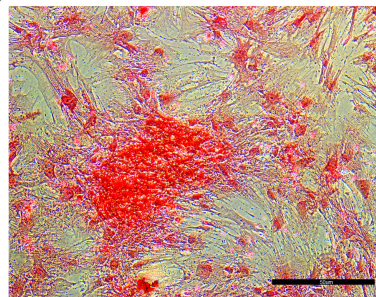

Supplement: Supplementary file 1 — Characteristics of ADSCs. (PDF 1563 kb) [file 13287_2018_1029_MOESM1_ESM.pdf]
