# Supplementary material for: Inhibition of autophagy promoted high glucose/ROS-mediated apoptosis in ADSCs
Source: Stem Cell Res Ther. 2018 Oct 25;9:289. doi: 10.1186/s13287-018-1029-4 (PMC6203262; doi:10.1186/s13287-018-1029-4)
Supplement: Supplementary file 2 — Supplementary materials and methods. (PDF 56 kb) [file 13287_2018_1029_MOESM2_ESM.pdf]

Flow cytometric analysis

Cell surface marker expression was examined as follows: fluorochrome-conjugated anti-human CD31-FITC, CD34-PE, CD90-FITC, CD105-APC, CD44-PE, and CD106-APC antibodies. These antibodies were purchased from BD Pharmingen (San Diego, CA, USA) and used in accordance with the instructions of the manufacturer. Non-specific staining was controlled by the use of isotype-matched antibodies. ADSC suspensions were incubated with the primary antibodies (1:50) for 30 min at room temperature. After incubation, the cells were washed twice with PBS and analyzed using a flow cytometer (BD Biosciences, Franklin Lakes, NJ, USA).

Osteogenic differentiation

ADSCs at passages 3-5 were seeded in six-well plates that were pre-coated with a 0.1% gelatin solution (Cyagen Bioscience, Inc., Guangzhou, China) at a density of 10^5 cells per well and allowed to reach 80-90 % confluence. Osteogenic differentiation was achieved by using a basic medium containing 0.1 μmol/l dexamethasone, 50 μmol/l ascorbic acid, and 10 mmol/l β-glycerophosphate for 3 weeks (Cyagen Bioscience, Inc., Guangzhou, China). Medium was replaced every 3 days. At the endpoint, cells were fixed with 4% paraformaldehyde in PBS for 15 min at room temperature and stained with alizarin red S following the manufacturers' instructions, to assess osteogenic differentiation. Specific stained ADSCs were documented under the Olympus IX71 light microscope (Olympus, Tokyo, Japan).
